# Contextual barriers to PrEP uptake and continuation among young Black gay and bisexual men who have sex with men living in the South

**DOI:** 10.1371/journal.pone.0334285

**Published:** 2025-10-10

**Authors:** Maira Sohail, Andrew O. Westfall, Ashleigh Chiedo, Bernadette Johnson, K. Rivet Amico, Patrick S. Sullivan, Jeanne Marrazzo, Janet M. Turan, Michael J. Mugavero, Latesha Elopre

**Affiliations:** 1 Department of Medicine, University of Alabama, Birmingham, Alabama, United States of America; 2 Department of Medicine, Ohio University Heritage College of Osteopathic Medicine, Athens, Ohio, United States of America; 3 School of Public Health, University of Michigan, Ann Arbor, Michigan, United States of America; 4 Rollins School of Public Health, Emory University, Atlanta, Georgia, United States of America; 5 School of Public Health, University of Alabama, Birmingham, Alabama, United States of America; 6 Department of Public Health, School of Medicine, Koç University, Sarıyer/İstanbul, Turkey; Beth Israel Deaconess Medical Center/Harvard Medical School, UNITED STATES OF AMERICA

## Abstract

Young, Black Gay, and Bisexual men who have sex with men (YBGBM) are disproportionately impacted by HIV, especially in Southern United States. We conducted a cross-sectional survey (Feb19-Mar20). Eligibility criteria were self-reported age 16–29 years, HIV-negative, Black race, and cis-gender male. We assessed associations between demographics, religiosity, intersectional stigma, and pre-exposure prophylaxis (PrEP) use (never, previous or current) among YBGBM in Alabama. Univariate and multivariable multinomial logistic regression models were fit with factors selected a priori, guided by a conceptual framework including individual-, interpersonal- and structural-level barriers to PrEP. 305 participants completed surveys (median age 24, 75% employed, 32% lacked personal transportation, and 41% reported annual incomes < $15,000). Compared to never PrEP use (n = 219), factors associated with current PrEP use (n = 51) included: ≥ college degree [AOR (95% CI): 5.48 (2.05, 14.62)], friends’ social support [AOR (95% CI): 1.33 (1.00, 1.52)], perceived HIV risk [AOR (95% CI): 1.27 (1.14, 1.42)], and PrEP knowledge [AOR (95% CI): 1.42 (1.23, 1.65)] AND factors associated with previous PrEP use (n = 35) included: depression [AOR (95% CI): 3.08 (1.34, 7.09)], condom use less than all the time [AOR (95% CI): 11.98 (1.52, 94.41)], intrinsic religiosity [AOR (95% CI): 0.77 (0.68, 0.88)], stable housing [AOR (95% CI): 0.30 (0.11, 0.81)], perceived sexual stigma [AOR (95% CI): 0.84 (0.75, 0.94)], and perceived HIV risk [AOR (95% CI): 1.18 (1.05, 1.33)]. YBGBM face distinct challenges with engagement in HIV prevention services and further investigation is needed to understand individual, interpersonal as well as structural-level factors that may mediate the ability to utilize PrEP services. Tailored multilevel strategies are urgently needed to improve PrEP uptake and persistence in YBGBM.

## Introduction

In the United States (U.S.), gay and bisexual men who have sex with men (GBM) accounted for 70% of newly acquired HIV infections in 2022 [1,2], despite accounting for only a small proportion of the total U.S. population [3]. Black GBM are one of the most likely group to acquire HIV and constitute 34% of all new HIV diagnoses among GBM [1,2]. Particularly, Young, Black Gay and Bisexual men who have sex with men (YBGBM) who are three times more likely to be diagnosed with HIV compared with white GBM and are not experiencing declines in HIV rates [3–6]. The U.S. Food and Drug Administration (FDA) approved the use of Truvada as pre-exposure prophylaxis (PrEP) on July 16^th^, 2012 for HIV-negative individuals who are more likely to be diagnosed with HIV due to epidemiologic risk factors including community rates [7]. In the following years, PrEP options expanded to include another oral (Descovy) and an injectable (Apretude) for HIV prevention [8]. While PrEP has proven to be 99% effective in preventing HIV acquisition with consistent use in all populations, uptake of PrEP among YBGBM still remains low, especially in the Southern U.S. where the need is greatest [8–11].

In the Southern U.S., an area disproportionally impacted by the HIV epidemic, there is a dearth of research comprehensively assessing contextual barriers to PrEP use among YBGBM [12,13]. A commonly reported reason for lower PrEP uptake among YBGBM is intersectional stigma [14]. Where stigma is defined as “a set of negative and unfair beliefs that a society or group of people have about something”, intersectional stigma is when multiple stigmas within an individual occur at the same time and have the power to influence each other [15,16]. In addition to intersectional stigma, other factors, such as structural barriers occurring at individual-level such as lack of medical insurance [17], interpersonal such as norms around sexual practices that influence stigma [18], and societal levels such as lack of healthcare access, have also been reported as reasons for lower PrEP uptake [19,20]. Interventions addressing these contextual barriers to PrEP uptake are urgently needed, especially in the South [21].

**Fig 1:**
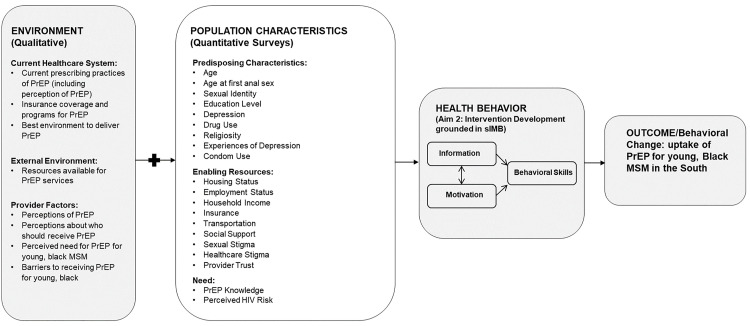
Conceptual model- conceptual model – grounded in andersen behavioral model with situated Information, Motivation, and Behavioral (sIMB) constructs for intervention development.

We conducted a qualitative study among YBGBM in Alabama to explore barriers to utilization of PrEP and found lower perceived HIV risk, lower prioritization and interest in PrEP use, intersectional stigma related to being Black, gay and living in the South, lack of information on how to access PrEP, and negative beliefs around PrEP use [22]. However, to understand the generalizability of such findings, quantitative research is needed to support intervention development tailored for YBGBM living in the South. Fig 1 depicts the project’s broader study outline, which aimed to inform an intervention development (health behavior box) to improve PrEP uptake among YBGBM. The current study is the quantitative part (population characteristics box) of a sequential mixed methods study, where previously conducted in-depth interviews (environment box) informed the surveys completed in this study. In addition to the in-depth interviews conducted as a part of this study, some of the variables assessed within the quantitative arm of the study were also informed by another pilot study which conducted qualitative interviews. Some of the variables assessed in the quantitative arm of this study were informed by prior qualitative research, a phenomenological exploration of sexual health among Southern, Black MSM [23]. Informed by our in-depth interviews, the objective of this study was to quantitatively assess factors that impact PrEP use among YBGBM, evaluating differences between current PrEP users, previous PrEP users (individuals that were on PrEP at some point but decided to discontinue), and those who have never used PrEP.

The study was grounded in the Andersen Behavioral Model framework, followed by an individual-level intervention driven by the situated Information, Motivation, and Behavioral skill theoretical framework (sIMB) [24,25]. The Andersen Behavioral Model framework postulates that individual (ex. age or sexual identity), societal (ex. sexual or healthcare stigma), and contextual (ex. policies) factors impact healthcare utilization, whereas the sIMB proposes that information (ex. PrEP knowledge), motivation (ex. Perceived HIV risk) and behavioral skills (ex. Condom use all the time) within the cultural and situational context in which they occur are necessary for initiating and maintaining behavioral change. The Andersen Behavioral Model framework has a potential to influence PrEP use at multiple socio-ecologic levels, as the domains including population characteristics (explored in this paper with predisposing characteristics, enabling resources, and need as constructs), health behavior, and environment (i.e., evaluating individual, community-, and policy-level factors that align with these domains).The Andersen Behavioral Model framework provides a granular understanding of how multi-level contextual factors impact utilization of health services, such as PrEP care, whereas the sIMB allows for evaluation of the core determinants for adopting health behaviors associated with PrEP uptake.

Previous literature has suggested facilitators to PrEP utilization, including PrEP education and awareness as well as strategies to actively address PrEP-related barriers, such as PrEP care discrimination, perceived need for PrEP prevention, PrEP-related or homosexuality-related stigma, social or peer support, and PrEP-related costs [26–28]. Therefore, the goal of this study was to gain a better understanding of contextual factors that may pose as facilitators or barriers in the uptake of PrEP among YBGBM living in Alabama. Gaining an understanding of the barriers to PrEP uptake will help towards developing a tailored intervention for YBGBM living in the Southern U.S. aimed at increasing PrEP uptake in this population. Moreover, prior studies, including in-depth interviews conducted by our research team, report that sexual identity, condom use, PrEP knowledge, perceived sexual stigma, and provider mistrust as factors impacting PrEP willingness among BMSM [23,29,30]. Using this knowledge and that gained through our IDIs concepts, we also assessed factors associated with PrEP willingness among the “never PrEP users”.

## Methods

### Study design

This cross-sectional study consisted of a self-administered, electronic survey distributed to YBGBM living in Alabama. The survey focused on evaluating the population characteristics that influence PrEP use and included the following Andersen Behavioral Model constructs: predisposing characteristics (attributes not directly related to the health-behavior that may still influence utilization), enabling factors [factors that can either service as barriers or facilitators for service utilization [31]], and needs (factors that influence perceived need for the service).

### Recruitment and data collection

Study recruitment was primarily through chain-referral and social media outlets. The Jack’d, a popular social networking application among YBGBM, was used as a medium for direct messaging campaigns and banner advertisements. In addition to Jack’d, recruitment was done through “The Game Changer Project” (PS15–1509), which is a Centers for Disease Control and Prevention-funded program for providing comprehensive HIV prevention and care services to GBM of color. AIDS Alabama, a Community Based Organization, was the lead agency for this project and through the existing partnership between AIDS Alabama and University of Alabama at Birmingham Center for AIDS Research, additional recruitment was carried out by Black GBM. The eligibility criteria included, 1) self-reported age 16–29 years; 2) black race; 3) HIV-negative infection status; 4) male (based on gender and sex at birth) with self-reported past (six months) sex with other men. Soon after recruitment (February 2019 to March 2020), eligible YBGBM completed a one-time, self-administered, electronic survey, which lasted about 20–30 minutes. This method of data collection has shown to be successful in collecting data on sensitive topics [32,33]. Each participant was compensated with $50 for their time and participation. The study was approved by the University of Alabama at Birmingham Institutional Review Board (IRB # 00000726). Consent was informed and electronically signed by the study participants. Consent was waived for minors by the IRB.

### Outcome variables

The survey data was accessed on January 20^th^, 2021, after all surveys were completed and uploaded to the database. The authors did not have access to information that could identify individual participants during or after data collection. The primary outcome, PrEP use, was self-reported and was categorized as current PrEP user, previous PrEP user, and never PrEP user. Additionally, the willingness to starting PrEP among never PrEP users was also captured, PrEP was described as a medication that reduces the chances of getting HIV. This was followed by assessing PrEP willingness in this sub-group with response options, “yes”, “no”, and “don’t know”.

### Predictor variables

Demographics were captured at the time of survey completion and included age and age at first anal sex as continuous variables and sexual identity categorized as gay and other (bisexual or asexual); housing status categorized as stable housing and unstable housing; education categorized as high school or less, some college, and college degree; employment status categorized as employed and unemployed; household income (annual) categorized as <$15,000, $15–30,000, $30–50,000, and>$50,000; insurance status categorized as insured/Medicaid and uninsured; transportation categorized as personal transportation and public/no transportation; drug use categorized as current use and no use; and condom use categorized as all the time, less than all the time, and missing (due to a substantial amount of missing data on condom use leading to sample loss in the multivariable analysis, a separate category for missing observations was made). A sensitivity analysis was conducted in which individuals with missing data on condom use were excluded (sample size = 254). The findings from this analysis were very similar to the model with three-category condom use (Table 2), therefore, a decision was made to include missing as a separate category to avoid loss of substantial sample size.

**Table 1:** Characteristics of study population by reported PrEP use among MSM in Alabama, 2019-2020 (n = 305).

Characteristics	Never PrEP Use N = 219	Previous PrEP Use N = 35	Current PrEP Use N = 51	p-value
Age^‡^	24 (21, 27)	25 (24, 27)	24 (22, 27)	0.1931
Age at First Anal Sex^‡^	17 (15, 18)	16 (13, 21)	17 (15, 18)	0.5187
Sexual Identity^†^				0.2736
Gay	165 (76)	27 (77)	44 (86)	
Other (Bisexual/Asexual)	53 (24)	8 (23)	7 (14)	
Housing Status^†^				**<0.0001**
Stable Housing	191 (87)	18 (51)	46 (90)	
Unstable	28 (13)	17 (49)	5 (10)	
Education^†^				**0.0032**
High school or less	92 (42)	18 (51)	12 (24)	
Some College	93 (42)	9 (26)	20 (39)	
Degree	34 (16)	8 (23)	19 (37)	
Employment Status^†^				0.6167
Employed	165 (75)	25 (71)	41 (80)	
Unemployed	54 (25)	10 (29)	10 (20)	
Household Income^†^				**0.0438**
< $15,000	82 (41)	22 (63)	14 (30)	
$15, 000-30,000	65 (32)	10 (29)	18 (38)	
$30, 000-50,000	40 (20)	1 (3)	11 (23)	
> $50,000	15 (7)	2 (6)	4 (9)	
Insurance Status^†^				**0.0379**
Insured/Medicaid	155 (71)	31 (89)	42 (82)	
Uninsured	64 (29)	4 (11)	9 (18)	
Transportation^†^				**0.0410**
Personal	148 (68)	17 (49)	38 (75)	
Public or None	71 (32)	18 (51)	13 (25)	
Drug Use^†^				0.0629
Current Use	112 (51)	10 (29)	23 (46)	
No Use	106 (49)	24 (71)	27 (54)	
Depression				**0.0009**
Yes (mild/moderate/severe)	76 (35)	23 (66)	13 (26)	
No	143 (65)	12 (34)	37 (74)	
Condom Use^†^				**0.0327**
All of the time	62 (28)	1 (3)	12 (24)	
Less than all of the time	123 (56)	27 (77)	36 (70)	
Missing	34 (16)	7 (20)	3 (6)	
Perceived HIV Risk^‡^	2 (0, 5)	4 (3, 5)	5 (2, 8)	**<0.0001**
Religiosity^‡^				
Organizational	2 (0, 5)	3 (2, 4)	3 (2, 4)	0.2762
Non-organizational	2 (1, 4)	2 (2, 3)	2 (1, 4)	0.8605
Intrinsic	11 (9, 13)	7 (6, 9)	11 (8, 14)	**0.0008**
Social Support^‡^				
Significant Other	5 (4, 7)	4 (3, 6)	6 (5, 7)	**0.0030**
Family	5 (4, 7)	4 (3, 6)	6 (3, 7)	**0.0315**
Friends	6 (4, 7)	4 (3, 6)	6 (5, 7)	**0.0013**
Total	5 (4, 6)	4 (3, 5)	6 (5, 7)	**0.0016**
PrEP Knowledge^‡^	6 (3, 8)	6 (5, 8)	9 (7, 10)	**<0.0001**
Experiences of Discrimination^‡^	0 (0, 5)	2 (0, 5)	3 (0, 6)	0.8818
Sexual Stigma^‡^				
Perceived	7 (4, 11)	4 (1, 6)	8 (5, 10)	**0.0003**
Enacted	3 (0, 6)	5 (0, 7)	3 (0, 6)	0.5257
Total	11 (6, 16)	11 (3, 13)	10 (7, 18)	0.1198
Healthcare Stigma^‡^	3 (3, 6)	6 (3, 6)	3 (3, 6)	**0.0024**
Provider Trust^‡^	9 (9, 12)	7 (6, 9)	9 (9, 11)	**0.0015**

^a^^‡^Median (interquartile range); ^†^ N (%).

^b^P-values from separate univariate multinomial logistic regression models.

^c^Bold denotes significance.

**Table 2:** Logistic regression models assessing factors associated with PrEP use among MSM in Alabama, 2019-2020 (Reference = Never PrEP users, n = 305).

Independent Variable	Current PrEP Use		Previous PrEP Use	
	OR (95% CI)	AOR (95% CI)	OR (95% CI)	AOR (95% CI)
	**Predisposing Factors**			
Age	1.06 (0.96, 1.17)	0.98 (0.87, 1.10)	1.09 (0.97, 1.23)	1.14 (0.99, 1.32)
Sexual Identity				
Gay/same Gender vs Other	2.02 (0.86, 4.75)	2.08 (0.79, 5.42)	1.08 (0.47, 2.53)	0.71 (0.27, 1.87)
Education				
Some College vs High school	1.65 (0.76, 3.57)	2.10 (0.90, 4.90)	0.50 (0.21, 1.16)	0.70 (0.27, 1.83)
Degree vs High school	**4.28 (1.88, 9.76)**	**5.48 (2.05, 14.62)**	1.20 (0.48, 3.02)	1.27 (0.42, 3.78)
Depression				
Yes vs No	0.66 (0.33, 1.32)	0.49 (0.23, 1.05)	**3.61 (1.70, 7.65)**	**3.08 (1.34, 7.09)**
Condom Use (ref all of the time)				
Less than all of the time	1.51 (0.74, 3.11)	1.90 (0.86, 4.19)	**13.61 (1.81, 102.50)**	**11.98 (1.52, 94.41)**
Missing	0.46 (0.12, 1.73)	0.43 (0.09, 2.12)	**12.77 (1.51, 108.12)**	**9.59 (1.07, 86.28)**
Religiosity				
Non-organizational	1.01 (0.84, 1.22)	1.00 (0.81, 1.23)	1.06 (0.86, 1.31)	1.28 (0.96, 1.69)
Intrinsic	0.89, 1.06)	0.95 (0.86, 1.05)	**0.83 (0.75, 0.91)**	**0.77 (0.68, 0.88)**
	**Enabling Factors**			
Housing Status				
Stable vs Unstable	1.35 (0.49, 3.68)	1.13 (0.37, 3.45)	**0.16 (0.07, 0.34)**	**0.30 (0.11, 0.81)**
Insurance Status				
Insured/Medicaid vs Uninsured	1.93 (0.89, 4.19)	1.89 (0.85, 4.18)	3.20 (1.09, 9.43)	2.15 (0.68, 6.74)
Social Support				
Friends	**1.23 (1.00, 1.52)**	**1.33 (1.03, 1.72)**	**0.76 (0.62, 0.92)**	1.15 (0.85, 1.54)
Sexual Stigma				
Perceived	1.01 (0.94, 1.08)	1.00 (0.93, 1.08)	**0.82 (0.74, 0.90)**	**0.84 (0.75, 0.94)**
Healthcare Stigma	1.05 (0.87, 1.28)	1.14 (0.91, 1.42)	**1.42 (1.17, 1.73)**	1.22 (0.96, 1.55)
Provider Trust	1.00 (0.88, 1.15)	0.94 (0.80, 1.10)	**0.77 (0.67, 0.89)**	0.85 (0.70, 1.02)
	**Needs Factors**			
Perceived HIV Risk	**1.26 (1.14, 1.40)**	**1.27 (1.14, 1.42)**	**1.17 (1.04, 1.32)**	**1.18 (1.05, 1.33)**
PrEP Knowledge	**1.43 (1.23, 1.65)**	**1.42 (1.23, 1.65)**	1.10 (0.98, 1.24)	1.11 (0.98, 1.26)

^a^Bold denotes p < 0.05.

Social determinants were mapped to the conceptual framework and assessed predisposing factors, enabling factors, and needs. Predisposing factors included age, age at first anal sex, sexual identity, education, depression, drug use, religiosity, experiences of discrimination, and condom use. Enabling factors included housing status, employment status, household income, insurance, transportation, social support, sexual stigma, healthcare stigma, and provider trust. PrEP knowledge and perceived HIV risk were within the needs construct. All variables were captured using validated measures, with the exception of PrEP knowledge, which was captured using a non-validated measure derived from our previous studies with eligible PrEP users [22,23]. Depression was assessed using the patient health questionnaire (PHQ-9) [34], a 9-item scale with a score ranging from 0–27 and was categorized as no depression (score 0–4) and mild/moderate/severe depression (score 5–27). Religiosity was captured using the DUREL (Duke University Religion Index) [35], a 5-item scale with three subscales, organizational religious activity (score range 0–5), for example “How often do you attend church or other religious meetings?”; non-organizational religious activity (score range 0–5), for example, “ How often do you spend time in private religious activities, such as prayer, meditation, or Bible study?”; and intrinsic religious activity (score range 0–14), for example, “In my life, I experience the presence of the Divine (i.e., God)”. Social support was captured using the Multidimensional Scale of Perceived Social Support [36], a 12-item scale with four categories (each category with a score range 1–28), which included social support from significant other, from friends, and from family; a total social support score was also calculated summing the scores from all 12 items (Score range 1–84). Social support for this study was categorized as social support from family, friends and significant other and a total score. Discrimination was measured using an 11-item subscale of the Experiences of Discrimination Scale [37]. The answer to each of the 11 items denotes the frequency of experiences of each type of discrimination and therefore, a higher score indicated greater discrimination experiences. Healthcare stigma was measured by using six questions; three questions on enacted stigma related to race and three questions on enacted stigma related to sexual orientation [38]. For example, “I have been ignored by health care providers because of my race” or “… because of my sexual orientation”; each question’s response score ranged from 1–4. Provider trust was measured using three questions, for example, “I trust health care providers are giving me the best available treatment” and each question’s response score ranged from 1–4 [38]. Perceived HIV risk was assessed using an ordinal scale; a higher score denoted greater perceived HIV risk. PrEP knowledge was based on a set of questions with true and false options. A sexual stigma scale developed for Lesbian, Bisexual, and queer women was adapted for GBM [39]. The scale measured five questions on perceived stigma, for example, “How often have you had to pretend that you are straight in order to be accepted?”; seven questions on enacted stigma, for example, “How often have you been harassed by the police for being GBM?”; and the responses for each question were captured using a 4-point Likert-type scale including never, once or twice, a few times, many times. A total score for sexual stigma was also calculated by combining the scores for perceived and enacted stigma.

### Statistical analysis

Descriptive statistics of associations with PrEP use were reported for predictor variables. For the demographics, median and interquartile ranges were reported for continuous variables and frequencies and proportions were reported for continuous variables. For categorical predictor variables, median and interquartile ranges were reported for each category within each variable. Univariate multinomial logistic regression models were fit for current PrEP use and previous PrEP use, using never PrEP use as the reference to calculate crude odds ratios (OR). For the multivariable analysis, a single multivariable multinomial logistic regression model with generalized logit link function was initially fit to calculate adjusted ORs (AOR) and their accompanying 95% confidence intervals (CI) for current PrEP use and previous PrEP use, using never PrEP use as the reference. However, due to missing values within variables and having too many variables in a single model, the final analysis was divided into three models (predisposing, enabling, and needs). For each of the three models, the variables that did not suggest statistically significant association were ranked from most to least important in relevance to the current study and were then removed one-by-one until adequate models were achieved. The predisposing model removed experience of discrimination (missingness in addition to non-significance), current drug use, age at first anal sex, and organizational religiosity, in the same order and the final model consisted of age, sexual identity, education, depression, condom use, and religiosity (non-organizational and intrinsic). The enabling model removed transportation, employment status, household income, social support from family, enacted sexual stigma (collinearity in addition to non-significance), and social support from significant others, in the same order and the final model contained housing status, insurance status, social support (friends), sexual stigma (perceived), healthcare stigma, and provider trust. No variables were removed from the needs model which had perceived HIV risk and PrEP knowledge. For the secondary analysis, PrEP willingness (“yes”) was assessed among never PrEP users using logistic regression models using a combined category of “no/don’t know” as the reference. All analyses were carried out in SAS 9.4 (SAS Institute Inc).

## Results

A total of 305 eligible individuals completed the survey and were included in the analysis (current PrEP use: 16%; previous PrEP use: 12%; never PrEP: 72%). The median age of participants was 24 years, and most were employed (75%) and had insurance/Medicaid (75%). Of the 219 individuals who reported never PrEP use, data on only 215 participants on PrEP willingness (Yes: 67%; No/don’t know: 33%) could be analyzed due to missing response from three participants. The bivariate analysis (Table 1) showed that current PrEP users were more likely to have a college degree, report higher social support from a significant other and family as well as overall social support, and report higher PrEP knowledge than previous and never PrEP users. Compared to never and current PrEP users, previous PrEP users were less likely to have stable housing, less likely to use condoms all the time, report lower intrinsic religiosity (or spirituality), report lower perceived and enacted sexual stigma, report lower provider trust and were more likely to report depression and healthcare stigma. Never PrEP users had lower perceived HIV risk and reported less experiences of discrimination than current and previous PrEP users.

The multivariable model assessing predisposing factors showed that current PrEP users had higher odds of having a college degree vs. high school [AOR (95% CI): 5.48 (2.05, 14.62)] than never PrEP user, whereas previous PrEP users had higher odds of reporting depression [AOR (95% CI): 3.08 (1.34, 7.09)] and use condom less than all the time [AOR (95% CI): 11.98 (1.52, 94.41)] and had lower odds of intrinsic religiosity [AOR (95% CI): 0.77 (0.68, 0.88)] than never PrEP users (Table 2). Among the model with enabling factors, current PrEP users had higher odds of social support from friends [AOR (95% CI): 1.33 (1.00, 1.52)], whereas previous PrEP users had lower odds of having stable housing [AOR (95% CI): 0.30 (0.11, 0.81)] and perceived sexual stigma [AOR (95% CI): 0.84 (0.75, 0.94)] and provider trust [AOR (95% CI): 0.77 (0.67, 0.89)] than never PrEP users. Lastly, among the model with needs, current PrEP users had higher odds of perceived HIV risk [AOR (95% CI): 1.27 (1.14, 1.42)] and PrEP knowledge [AOR (95% CI): 1.42 (1.23, 1.65)] than never PrEP user, whereas previous PrEP users had higher odds of perceived HIV risk [AOR (95% CI): 1.17 (1.04, 1.32)] than never PrEP users.

Variables included in the multivariable analysis assessing PrEP willingness among never PrEP users (n = 219) included sexual identity, condom use, intrinsic religiosity, social support from friends, PrEP knowledge, perceived sexual stigma, and provider trust. The results showed that gay versus other sexual identification [AOR (95% CI): 3.02 (1.17, 7.79)] and those with higher PrEP knowledge [AOR (95% CI): 1.14 (1.03, 1.26] had higher odds of PrEP willingness (Table 3).

**Table 3:** Logistic regression models assessing factors associated with PrEP willingness among never PrEP users, (n = 219).

Independent Variable	PrEP Willingness	
	OR (95% CI)	AOR (95% CI)
Sexual Identity		
Gay vs Other	**2.42 (1.28, 4.57)**	**2.60 (1.25, 5.41)**
Condom Use (ref all of the time)		
Less than all of the time	1.40 (0.72, 2.72)	1.60 (0.72, 3.57)
Missing	**0.36 (0.15, 0.85)**	0.48 (0.18, 1.27)
Religiosity		
Intrinsic	**1.11 (1.03, 1.20)**	1.07 (0.97, 1.18)
Social Support		
Friends	**1.28 (1.08, 1.51)**	1.10 (0.88, 1.37)
PrEP Knowledge	**1.19 (1.09, 1.30)**	**1.14 (1.03, 1.26)**
Sexual Stigma		
Perceived	**1.12 (1.05, 1.20)**	1.06 (0.99, 1.15)
Enacted	1.0 (0.9, 1.1)	–
Provider Trust	**1.18 (1.05, 1.33)**	1.16 (0.99, 1.36)

^a^Bold denotes significance <0.05.

## Discussion

Findings from this study indicate that among YBGBM, who are often marginalized due to their multiple stigmatized identities, there are critical differences among current PrEP users, previous PrEP users, and never PrEP users in relation to predisposing factors, enabling factors, and perceived need. Greater understanding of these factors may inform development of more individualized, nuanced and tailored interventions for YBGBM who are in different stages of the PrEP care continuum. Current PrEP and previous PrEP users were more educated and perceived their HIV risk to be higher than never PrEP users, which accounted for 72% of the total study population. Interestingly, although our study sample predominantly comprised of never PrEP users, majority of these individuals reported willingness to use PrEP in the next three months. Noteworthy, those who never used PrEP and reported PrEP use willingness also reported higher PrEP knowledge. Moreover, we were unable to assess why previous PrEP users were no longer on PrEP in this study, which is a critical area that should be explored in future research.

We found that current PrEP users were more likely to have a college degree and to have higher social support from friends. While not many studies among YBGBM have directly examined the impact of social support from friends on PrEP usage, studies conducted among broader MSM groups and transgender population have shown social support from friends to encourage PrEP utilization [40]. Additionally, higher level of education among YBGBM has shown to be associated with higher PrEP awareness, usage, and adherence, and may also suggest an increased understanding of the need for PrEP within an individual, which may also relate to their perception to HIV risk [21,41]. In line with this, our study also found never PrEP users have lower perceived HIV risk than current PrEP users. While lower perceived HIV risk may actually correspond to being at a lower likelihood of HIV acquisition in some cases, in others, it may be a result of the lack of understanding and effective messaging on the role of local HIV prevalence on actual risk for HIV acquisition, resulting in decreased prioritization of prevention strategies like PrEP. A study among Black MSM in California reported misconceptions associated with PrEP use, where participants perceived only risky sexual behaviors to be associated with a greater likelihood of HIV acquisition (e.g., condomless sex or multiple sex partners) and reasons to use [42]. Similarly, another study conducted in Tennessee among predominantly young, Black MSM exploring areas that could help increase PrEP uptake found that although participants had basic knowledge on PrEP, concerns were raised on PrEP being commonly labelled as a “promiscuity promoting” pill, which may in turn influence one’s perceived HIV risk and lead to discouragement for PrEP uptake [43]. This suggests a need for more interventions exploring ways to improve messaging to abate current misconceptions among YBGBM around HIV “risk” and PrEP need.

In addition to lower perceived HIV risk, we also found lower PrEP knowledge among never PrEP users. In line with this, a study exploring reasons for not utilizing PrEP among young Black MSM newly diagnosed with HIV in Alabama found that although majority of participants were aware of PrEP, lack of specific PrEP-related knowledge, such as how to access PrEP, etc. acted as a barrier to PrEP uptake [22]. Another study showed Black MSM have lower PrEP awareness compared to other race counterparts [44]. While we did not include young gay bisexual men from other races to draw a comparison, the evidence in all suggests that PrEP knowledge has an apparent influence on PrEP uptake among YBGBM and greater resources are needed to move beyond awareness campaigns to improve knowledge on PrEP.One effective strategy would be to provide clinicians with effective tools to aid them in accurately educating their patients about the important role of community HIV prevalence in HIV risk, while also clearing any PrEP-related misconceptions that may be contributing to the low PrEP uptake in this population.

In addition to lower PrEP knowledge and lower perceived HIV risk, we also found never PrEP users to have higher perceived sexual stigma and higher intrinsic religiosity. While previous literature has shown internalized sexual stigma or sexual orientation stigma to be associated with lower PrEP uptake among MSM at-risk for HIV acquisition [45,46], religiosity has not been evaluated extensively in relation to PrEP use among YBGBM; however, church attendance and spirituality have been investigated among people with HIV and has shown mixed findings on its protective versus harmful associations with HIV outcomes [47–51]. More work is needed in this area to fully understand the role of religiosity in HIV prevention efforts tailored towards Southern YBGBM.

Furthermore, our study in examining the unique, previous PrEP user population found this group to be distinguished from current and never PrEP users. Our findings indicated previous PrEP users experience higher healthcare stigma and have lower provider trust. While healthcare stigma and provider mistrust discouraging PrEP uptake among never PrEP users have consistently been noted in previous literature [45,52], more research is needed to determine if these factors are also associated with discontinuation of PrEP after initial uptake in this population.

Moreover, our study found greater PrEP willingness among those identifying as gay versus bisexual/asexual and those with higher PrEP knowledge. In line with our findings, a study young GBM in California, in which Black GBM constituted 25% of the total study sample, found that PrEP willingness was associated with perceived benefits of using PrEP [53]. Although not directly associated, perceived benefits of PrEP use may relate to higher PrEP knowledge, but more research is needed to further explore these relationships. In addition, we found it interesting that participants who identified as gay were more likely to report PrEP willingness than those who identified as bisexual or asexual, which calls for more work exploring perceptions among Black bisexual men to better understand unique barriers they may face.

PrEP uptake among YBGBM living in the South still remains a challenge that must be adequately met if we are to end the HIV epidemic in the U.S. Inequities among PrEP use are aberrant and widening, made clear by recent data showing that Black people have the lowest PrEP-to-Need ratios indicating they have the highest unmet need [54]. In our study sample of YBGBM, only 17% of participants reported current PrEP use [54]. These findings illustrate that multilevel barriers such as low perceived HIV risk, are not only influencing PrEP uptake but also persistence. Taking these findings together, our study indicates that PrEP uptake and persistence is a complex phenomenon, which is influenced by various social determinants occurring at the individual-, interpersonal-, and structural levels. In turn, overcoming these barriers will require multi-level interventions that are culturally appropriate and responsive to the needs of YBGBM living in the Deep South.

### Strengths and limitations

To our knowledge, this is the first study conducted among YBGBM that evaluated both intra-personal and inter-personal factors and their relationship to PrEP use among current, previous, and never PrEP users, as it relates to constructs within the Andersen Behavioral Model framework including predisposing and enabling factors as well as perceived personal need. In addition, previously conducted studies among YBGBM have mostly focused solely on factors contributing to PrEP uptake, leaving a gap in the literature on how previous PrEP users and potential PrEP users differ from each other demographically and socially. Additionally, this study represents findings from YBGBM living in the Southern US, where rates of PrEP use among YBGBM are very low, and where PrEP inequity by race is highest [54]. Moreover, this study used validated scales for measuring social determinants impacting PrEP use and examined associations with PrEP use using rigorous multivariable analytic methods. Our study also had several limitations. Our sample size was small and within the sample, the proportion of current PrEP users and previous PrEP users was quite small. Additionally, the reason behind discontinuing PrEP use among previous PrEP users was not examined. Moreover, all variables were self-reported by participants, and misclassification of PrEP use is possible. Lastly, intersectional stigma was perceived based on two scales capturing distinct stigmas within them, healthcare stigma and sexual stigma. Future research can build on these separate stigma scales and generate a single scale focused on capturing intersectional stigma within a population.

## Conclusions

Although YBGBM are 50% more likely to be infected with HIV than their white counterparts [55], PrEP use among this population still remains low, especially within the Southern U.S. Our study identified multi-level factors associated with current PrEP use and previous PrEP use (individuals that did start PrEP but discontinued), relative to never PrEP use among YBGBM. This study adds to the literature by evaluating contextual factors that may determine HIV preventive behaviors that have the potential to effectively end the epidemic. However, this will only be accomplished through development of culturally tailored interventions that act at multiple levels to address the many barriers faced by YBGBM living in the South.
